# Effect of Gear Ratio and Cadence on Gross Efficiency and Pedal Force Effectiveness during Multistage Graded Cycling Test Using a Road Racing Bicycle

**DOI:** 10.3390/sports11010005

**Published:** 2022-12-23

**Authors:** Mutsumi Kamba, Hisashi Naito, Hayao Ozaki, Shuichi Machida, Shizuo Katamoto

**Affiliations:** 1 Graduate School of Health and Sports Science, Juntendo University, 1-1 Hirakagakuendai, Inzai, Chiba 270-1695, Japan; 2 Faculty of Human Ecology, Wayo Woman’s University, 2-3-1 Konodai, Ichikawa, Chiba 272-8533, Japan; 3 School of Sport and Health Science, Tokaigakuen University, 21-233 Nishinohora, Ukigai, Miyoshi, Aichi 470-0207, Japan

**Keywords:** pedaling technique, pedaling strategies, mechanical efficiency, cycling

## Abstract

Gross efficiency (GE) and the index of pedal force effectiveness (IFE) are important factors that enhance cyclists’ performance; however, the effects of changing pedal force (gear ratio) and cadence on these indices while riding on a road racing bicycle are poorly investigated. This study aimed to examine the effect of changing gear ratio or cadence on GE and IFE using a road racing bicycle. Nine male cyclists completed graded submaximal cycling tests (five stages of 4 min submaximal cycling sessions with 1 min passive rest intervals). The work rate of each stage was determined using two principles: changing gear ratio at a fixed cadence and changing cadence at a fixed gear ratio. We determined GE and IFE using respiratory variables and pedal reaction forces, respectively. Increasing the gear ratio improved GE, and was associated with the IFE. Although increasing the cadence slightly improved GE from the initial level, the increased values then mostly maintained. IFE was almost stable even when cadence increased. Moreover, no significant correlation was observed between the changes in GE and IFE accompanied by increasing cadence. Our data indicate that an increasing gear ratio, but not cadence, may affect GE and IFE while riding on a road racing bicycle.

## 1. Introduction

During cycling competitions, it is important to maximize gross efficiency (GE), defined as the mechanical work rate relative to the total energy expenditure of a cyclist, to ensure the highest endurance [1]. Previous evidence suggests that the potential factors influencing GE are not only hardly modified indices, including environmental factors (e.g., ambient temperature and humidity) [2], but also relatively easily modified indices, including cyclist pedaling strategies [3,4,5,6]. Thus, many cycling athletes and their coaches seek optimal pedaling strategies to maximize GE. Pedal force (i.e., gear ratio) and cadence are two pedaling strategies that cyclists can change while riding, and how these factors affect GE has been examined. It was observed that increasing the pedal force at a fixed cadence gradually improved GE during an incremental cycling test [6,7]. Simultaneously, the ratio of the tangential force to the resultant force applied to the crank arm, referred to as the index of pedal force effectiveness (IFE), also increases as the gear ratio increases. Additionally, improvement in IFE was positively correlated with an increase in GE [6]. Since IFE is known as a parameter assessing the cyclist pedaling technique [3,4,8], it has been believed that a strategy to increase IFE also improves GE, and the training method for enhancing IFE has been used in previous training scenes and/or competitions [8].

Competitive road cyclists must control their cycling speed according to the road situation using not only the pedaling gear ratio and but also the cadence to maintain safe and efficient cycling. Moreover, as the bike used in track cycling has a fixed pedaling gear ratio, cyclists can control the cycling speed by only changing their cadence. Although many previous studies have focused on the effect of altering the gear ratio on GE and IFE, these practical views suggest the importance of evaluating the effect of changing cadence on GE and IFE. Several previous studies have confirmed that GE decreases as cadence increases at a fixed work rate; thus, it is thought that increasing cadence impairs GE [9]. However, these studies examined GE with changing cadence at a fixed work rate, thereby increasing cadence was accompanied by decreasing pedal force to maintain work rate. Accordingly, it is possible that the reduction of GE occurred due to the decreasing pedal force rather than the increasing cadence. Hence, the effect of changing cadence at a fixed pedal force on GE and IFE should be examined. Theoretically, the energy demand of the primary working muscle relative to the same absolute pedaling load per revolution is steady whenever the cadence is increased. However, the energy demand in various tissues other than the working muscle may also increase (e.g., postural stabilization) when demanding a higher work rate, suggesting that external work relative to the total energy cost, which does not account for effective pedal cranking, would be increased [10]. Collectively, it is currently unclear whether increasing cadence impairs GE.

Another concern is that most previous studies used GE and IFE with an electrobrake ergometer, which does not reflect real road racing bicycles [3,6,7,11]. Thus, whether the GE and IFE evaluated by these experimental ergometers can be applied to real training scenarios and/or competitions remains to be elucidated. That is, GE and IFE should be examined using a racer bike, which is used for real competitive races, with alterations in pedal force and cadence.

Therefore, this study aimed to investigate the effects of pedal force and cadence on GE and IFE using a road-racing bicycle. To test this, we examined two types of multistage graded submaximal cycling tests: changing pedal force (gear ratio) at a fixed cadence and changing cadence at a fixed gear ratio in well-trained college male cyclists.

## 2. Materials and Methods

### 2.1. Participants

Nine male national- and regional-level collegiate endurance cyclists volunteered to participate in this study. Their physical characteristics (means ± standard deviation) were age, 20.4 ± 1.7 years; height, 170.8 ± 4.7 cm; body weight, 64.1 ± 5.4 kg; and maximal oxygen uptake (*V*O_2_max), 64.9 ± 5.9 mL·kg^−1^·min^−1^. They had at least 3 years training experience (5.4 ± 1.5 years) and were all well-trained cyclists who had been training at least six days per week. All participants were informed of the possible risks associated with the experimental procedures prior to providing written consent. The study protocol was approved by the Research Ethics Review Board of Juntendo University (28-106), and the study was performed in accordance with the Declaration of Helsinki. 

### 2.2. Properties of the Experimental Bicycle

All measurements were conducted using a competition racer bike with a pedaling power meter (SGY-PM910V FC-6800, Pioneer, Tokyo) and a 3-roller bicycle trainer with a constant friction-loaded apparatus (AC-Magroller, Minoura, Japan). All participants rode the same experimental bike, which had 170 mm crank, wheels (Bontrager, Trek, USA), and tire (Topzio pro, Vittoria, Japan), in order to ensure uniform aerodynamic drag of the wheel and rolling resistance of the tire. The seat position was adjusted according to participants’ preferences. The free roller was specified for bicycle training, and the apparatus consisted of three rollers (diameter 100 mm, width 395 mm). Although the magnetic resistance was adjustable, with six levels of strength, we attached it to the rear roller position of the free roller at the first level. The resistance level was determined to adjust the target work rates in subsequent multistage cycling tests.

### 2.3. Multistage Graded Submaximal Cycling Test

**Figure 1:**
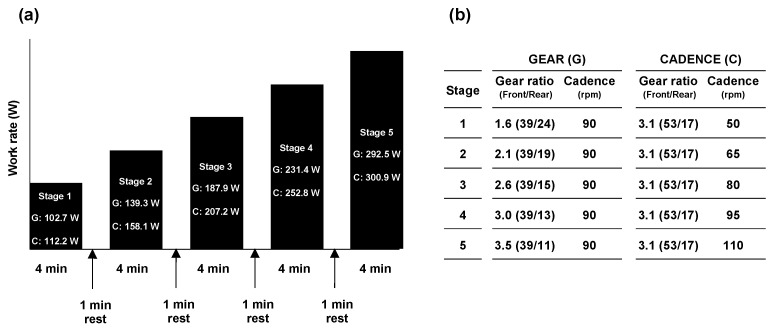
Overview of the experiment. (**a**) Experimental protocol for multistage graded cycling tests under two trial conditions. The work rate in each stage for the GEAR and CADENCE trials was indicated in the black box; (**b**) Experimental settings of gear ratio and cadence in each stage for the GEAR and CADENCE trials. G, GEAR trial; C, CADENCE trial.

All participants visited the laboratory three times throughout the experiment. During the initial visit, a familiarization session consisting of adjusting the saddle position of the aforementioned competition racer bike to match the participants’ preferences and practicing riding on a free bicycle roller (AC-Magroller) while wearing a face mask to collect respiratory gas for the exercise tests was performed. At least 24 h following the familiarization session, the participants visited the laboratory twice (main experiments) on different days. In the main experiments, participants performed two types of graded submaximal cycling tests while wearing a continuous heart rate (HR) monitor and face mask: increasing the work rate by changing the gear ratio at a fixed cadence (defined as ‘GEAR’ trial) and changing cadence at fixed gear ratio (defined as ‘CADENCE’ trial). The racer bike used gear ratio levels between 1.9 and 4.8 for this study; gear levels were changed by alternating the front and rear gears, which in turn altered the pedal force. In the GEAR trial, participants initially cycled with a gear ratio of 1.6 and a cadence of 90 rpm for 4 min on a free bicycle roller. Following a 1 min passive rest on the bicycle, cycling was restarted with a gear ratio of 0.5 higher than the initial gear ratio, but the cadence was maintained at 90 rpm. This 4 min cycling and 1 min rest session was repeated five times until the gear ratio was 3.5 (Figure 1). In the CADENCE trial, the participants performed a five-stage cycling test, increasing the cadence by 15 rpm in each stage, beginning at 50 rpm to 110 rpm with a fixed gear ratio of 3.1. During the graded cycling tests in both trials, the participants were asked to adjust their cadence with the sound of a metronome [12]. In this study, the absolute work rate in each stage during the graded submaximal cycling test differed between the GEAR and CADENCE trials because of the methodological limitations of using the racer bike. This experimental setting of gear ratio and cadence was determined based on a previous study [13], while considering the characteristics of road racing bicycles. During the cycling test, the HR and respiratory variables (oxygen uptake (*V*O_2_), carbon dioxide production (*V*CO_2_), and respiratory exchange ratio (RER)) were continuously measured throughout the experiment.

### 2.4. Measurements

**Figure 2:**
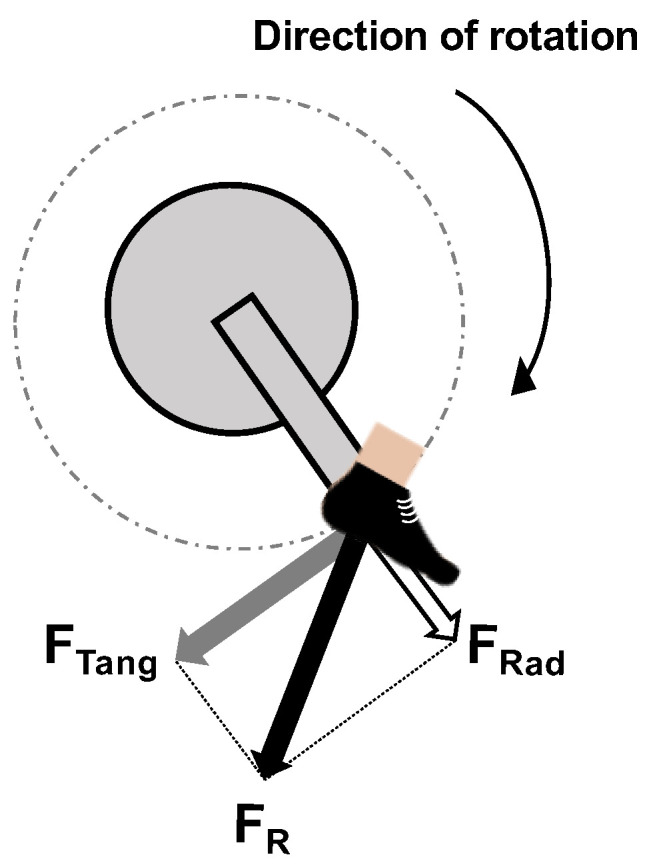
Principle of index of pedal force effectiveness. Resultant force (F_R_) applied in the plane of the crank; Tangential force (F_Tang_) tangential to the crank displacement (effective force) and radial force (F_Rad_) along the crank (ineffective force).

The HR was continuously measured during the test using a wireless HR sensor (30250 PowerCal ANT+, PowerTap, Japan). The cadence and pedal force (tangential and radial) were evaluated from the data obtained using a pedaling power meter (SGY-PM910V FC-6800, Pioneer, Tokyo). The pedaling power meter system has two streamlined strain gauge monitors that can detect the slightest tangent and radial force direction and force to the crank arms. In addition, the magnetic sensor detects the angle of rotation. This proprietary system detects the amount and direction of force at 12 different points during each pedaling stroke for each leg [14]. The validity of the pedaling power meter was confirmed by Sanders et al. in 2017 [15]. During the experiment, these data were continuously memorized by a cycle computer (SGX-CA500, Pioneer) attached to the experimental bike. Following the completion of the experiment, they were assessed using a dedicated analysis software (Cyclo-Sphere; Pioneer Corporation). Cyclo-Sphere calculates and displays the work rate (W), cadence (rpm), and IFE. According to previous studies, IFE was calculated as the ratio of the tangential force to the resultant force (Figure 2) [8]. Pedaling data were calculated from both the right and left legs, and their averaged values were used for further analysis.

*V*O_2_, *V*CO_2_, and RER were continuously monitored by collecting respiratory gas using an automatic gas analyzer (AE-300S, Minato Medical Science, Tokyo, Japan). Prior to each experiment, the flow sensor was calibrated using a 2 L syringe, and the O_2_ and CO_2_ sensors were calibrated using calibration gas (O_2_ 20.72%, N_2_ balance; O_2_ 15.09%, CO_2_ 5.00%, N_2_ balance, Sumitomo Seika Chemicals, Japan). The respiratory data averaged every 30 s were recorded, and the averaged values for the last 1 min during each stage of cycling were used for subsequent data analysis.

### 2.5. Data Analysis

We calculated the energy expenditure (EE) as follows: EE (J) = [(3.869 × *V*O_2_) + (1.195 × *V*CO_2_)] × (4.186/60) [16]. GE was calculated as work rate/EE × 100 (%) [16].

The Spearman’s correlation coefficient was used to examine the relationship between GE and IFE in each trial when the data were pooled. Briefly, in this study, these indices were assessed at five stages (GEAR trial:1.6, 2.1, 2.6, 3.1, and 3.5; CADENCE trial:50, 65, 80, 95, and 110 rpm) for nine participants; thus, the total number of plotted data would be 45. This analysis was based on previous studies that demonstrated that improvements in GE were closely associated with IFE during incremental cycling tests [3,6,17].

### 2.6. Statistical Analysis

Normality of the datasets obtained from each experiment was tested using the Shapiro–Wilk test; however, if equal variance failed, Friedman nonparametric and pairwise post hoc tests (Bonferroni) were used. Indeed, as equal variance failed in IFE, we analyzed it as a nonparametric dataset. One-way analysis of variance with Bonferroni’s post hoc test was used to compare the time course changes in *V*O_2_, *V*CO_2_, RER, and GE. For a graphical representation of these data, we represented the mean and individual values as solid and dotted lines, respectively. Microsoft Office Excel 2016 and Prism v. 9.0 (GraphPad Inc., La Jolla, CA, USA) were used for data processing and statistical analyses, respectively. Statistical significance was set at *p* < 0.05.

## 3. Results

**Figure 3:**
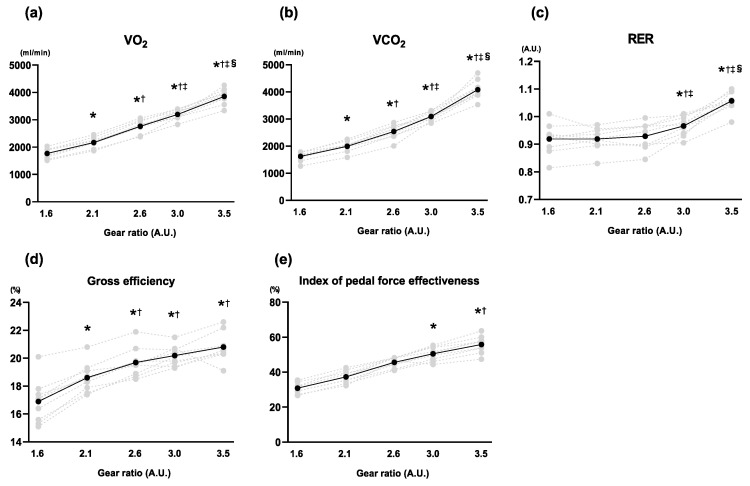
Changes in respiratory variables, gross efficiency, and index of pedal force effectiveness during multistage graded cycling test in the GEAR trial. Data representing mean and individual values are presented as solid and dotted lines, respectively. (**a**) Oxygen uptake; (**b**) Carbon dioxide production; (**c**) Respiratory exchange ratio; (**d**) Gross efficiency; (**e**) Index of pedal force effectiveness. * *p* < 0.01 versus 1.6; † *p* < 0.01 versus 2.1; ‡ *p* < 0.01 versus 2.6; § *p* < 0.01 versus 3.0. n = 9.

We first evaluated the effect of changing the gear ratio at a fixed cadence on the respiratory variables (*V*O_2_, *V*CO_2_, and RER), GE and IFE, during the five-stage graded cycling test (Figure 3). Both *V*O_2_ and *V*CO_2_ increased linearly as the gear ratio increased (Figure 3a,b). While RER at 2.1 and 2.6 of gear ratio (i.e., work rate = 139.9 and 187.3 W, respectively) did not significantly differ compared with the initial level (1.6 of gear ratio = 102.7 W of work rate), that was gradually increased when the gear ratio was more than 3.1 (Figure 3c). GE and IFE also gradually increased as the gear ratio increased (Figure 3d,e).

**Figure 4:**
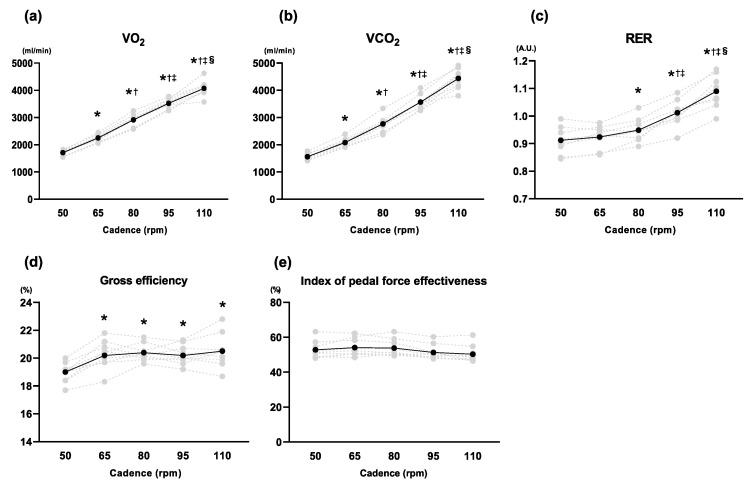
Changes in respiratory variables, gross efficiency, and index of pedal force effectiveness during multistage graded cycling test in the CADENCE trial. Data representing mean and individual values are presented as solid and dotted lines, respectively. (**a**) Oxygen uptake; (**b**) Carbon dioxide production; (**c**) Respiratory exchange ratio; (**d**) Gross efficiency; (**e**) Index of pedal force effectiveness. * *p* < 0.01 versus 50 rpm; † *p* < 0.01 versus 65 rpm; ‡ *p* < 0.01 versus 80 rpm; § *p* < 0.01 versus 95 rpm. n = 9.

Next, we assessed the effect of changing cadence at a fixed gear ratio on the respiratory variables, GE and IFE, during the five-stage graded cycling test (Figure 4). Similar to the GEAR trial, in the CADENCE trial, the respiratory variables increased with cadence (i.e., work rate) (Figure 4a–c). In contrast, although GE at a cadence of >65 rpm was significantly greater than that at 50 rpm, no significant changes were observed in the cadence between 65 and 110 bpm (Figure 4d). Moreover, IFE did not change throughout the CADENCE trial (Figure 4e).

**Figure 5:**
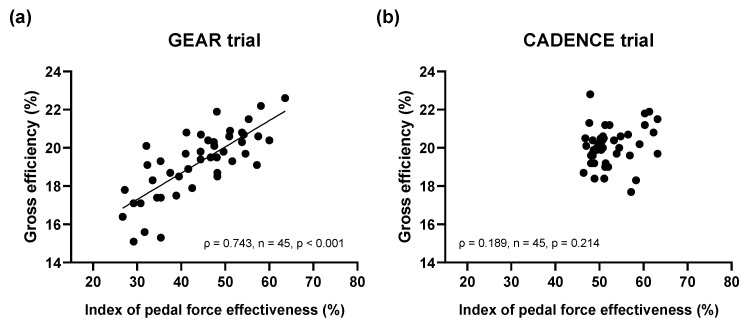
Relationship between index of pedal force effectiveness and gross efficiency. Plotted data represent all subjects (n = 9) at five stages. (**a**) GEAR trial; (**b**) CADENCE trial.

Further, we performed correlation coefficient analysis between GE and IFE in the GEAR and CADENCE trials (Figure 5). The GE was significantly correlated with IFE in the GEAR trial (Figure 5a), but not in the CADENCE trial (Figure 5b).

## 4. Discussion

To the best of our knowledge, this is the first study to measure GE and IFE during multistage graded cycling tests in a simulated real cycling situation by changing pedal force (gear ratio) and cadence using a road racing bicycle. As shown in Figure 3d,e, GE and IFE gradually increased with changing gear ratio at a fixed cadence (mean values of GE and IFE varied from 16.9–20.8% and 30.8–55.8%, respectively), and these parameters were closely associated. However, Figure 4d,e indicates that GE and IFE were relatively stable under the trial with changing cadence at a fixed gear ratio (mean values of GE and IFE were almost constant in the ranges of 19.0–20.5% and 50.3–52.8%, respectively). Moreover, a significant correlation between GE and IFE was not observed in this trial. Collectively, our results suggest that the patterns of alteration in GE and IFE accompanied by changing cadence are largely different from those with changing gear ratios.

Several previous studies have reported that the increase in work rate with increasing pedal force at fixed cadence improved GE and IFE during incremental cycling tests [6,7,11,18]. In accordance with previous studies, we confirmed that both GE and IFE increased linearly as the gear ratio increased in the GEAR trial. Although previous studies assessed GE and IFE using an electric cycle ergometer, we first confirmed that these physiological responses could be observed in a road racing bicycle in this study. Accordingly, this result suggests that GE and IFE may vary with changing gear ratio even in a real cycling situation.

Many previous studies have reported that increasing cadence decreases GE at a fixed work rate and concluded that increasing cadence has a deleterious effect on GE [9]. However, it must be acknowledged that increasing cadence is accompanied by a decreasing gear ratio when the work rate is maintained. As it has been demonstrated that changing gear ratio influences GE [9], the impairment of GE in previous studies might be related to the reduction in gear ratio rather than the increasing cadence. Therefore, we assessed GE and IFE during a graded cycling test with changing cadence at a fixed gear ratio. Interestingly, GE and IFE were almost stable in the CADENCE trial, despite the fact that the respiratory variables (*V*O_2_, *V*CO_2_, and RER) were increased, as in the GEAR trial. Based on physics theory, the energy demand of the primary work muscle relative to the same absolute pedaling load per revolution is steady when the cadence is increased. In contrast, an increasing work rate (exercise intensity) might be accompanied by increasing external energy costs that do not involve effective pedal force, such as stabilization in riding posture (recruiting previously inactive muscles) and changing muscle activation [10]. Moreover, it is assumed that various factors (e.g., air resistance, rolling resistance, alteration in the recruitment pattern of the muscle fiber type, and increased inertia force) induced by increasing the cadence could affect GE [10,19]. Based on these observations, we expected that increasing cadence would impair GE and IFE. However, our results suggest that increasing cadence itself did not affect GE and IFE, at least under a fixed gear ratio. It is further suggested that increasing cadence may not result in any energy expenditure other than effective cycling during the graded cycling test. Judging from the fact that the mean values of RER at Stage 5 (110 rpm) were >1.0, well-trained cyclists could maintain their effective pedaling even when the exercise intensity was severe.

Furthermore, a significant correlation between GE and IFE, which was observed in the GEAR trial, disappeared in the CADENCE trial. Cycling athletes and their coaches have often sought training strategies for enhancing IFE based on the concept that increasing IFE can also improve GE [8]. In contrast, Korff et al. [20] reported that a trial in which the participants were instructed to pull on the pedal during the upstroke induced significantly higher IFE, but significantly lower GE than other pedaling techniques, such as preferred pedaling, pedaling in circles, and pushing on the pedal during the downstroke. From these data, the authors concluded that IFE did not reflect GE across pedaling techniques, which may support our present results. Consequently, we speculate that enhancing IFE may not necessarily contribute to improving GE.

This study had some limitations. First, we set the cycling duration at each stage to 4 min. However, prolonged cycling, such as a competitive race (e.g., Tour de France), may induce central and peripheral fatigue, thus, GE and IFE would be impaired in this situation. Second, we used the IFE that is calculated from the radial force and the tangential force in the direction of pedal axle. We did not consider the lateral-medial force. The lateral-medial force may be lower than the other two forces, although we cannot deny that it influences the variables in this study. Third, we did not consider the involvement of anaerobic components in the calculation of GE. The exercise intensity at Stage 5 (RER > 1.0) might reach over the anaerobic threshold, over-estimating the GE. Fourth, because the experimental bike does not allow for a strictly variable work rate owing to the methodological limitations of the experimental bike, we set gear ratio and in each stage at absolutely same among participants in this study. Thus, the validity of the hypotheses derived from the results of this study should be confirmed by further studies that consider the relative exercise intensity of the participants. Moreover, we could not compare GE and IFE between the GEAR and CADENCE trials because the same work rate could not be set. Therefore, future studies should examine the various combination patterns with gear ratio and cadence at a fixed work rate to determine their optimal combination to maximize the cyclists’ performance. At last, since we recruited only well-trained competitive young male cyclists, it would be important, in future studies, to examine other cyclist populations, including world-class, junior, older adult, and female cyclists.

## 5. Conclusions

Our results demonstrated that increasing the pedal force at a fixed cadence linearly improved GE and IFE, even on a road racing bicycle. In contrast, GE and IFE remained almost stable despite an increase in cadence at a fixed pedal force. Although further studies are needed, our data raise the possibility that an increasing pedal force, but not cadence, influences GE and IFE while riding on a road racing bicycle. Moreover, not all training methods to improve IFE may reflect an enhanced GE. We expect these results to provide novel insights for reconsidering pedaling strategies in cycling and training methods to enhance cyclists’ performance.

## Data Availability

The data presented in this study are available on request from the corresponding author.
